# Epstein-Barr virus and mismatch repair deficiency status differ between oesophageal and gastric cancer: A large multi-centre study

**DOI:** 10.1016/j.ejca.2018.02.014

**Published:** 2018-05

**Authors:** L.C. Hewitt, I.Z. Inam, Y. Saito, T. Yoshikawa, A. Quaas, A. Hoelscher, E. Bollschweiler, G.E. Fazzi, V. Melotte, R.E. Langley, M. Nankivell, D. Cunningham, W. Allum, G.G. Hutchins, H.I. Grabsch

**Affiliations:** a Department of Pathology and GROW School for Oncology and Developmental Biology, Maastricht University Medical Center, Maastricht, The Netherlands; b Pathology and Tumour Biology, Leeds Institute of Cancer and Pathology, University of Leeds, Leeds, UK; c Department of Gastrointestinal Surgery, Kanagawa Cancer Center, Yokohama, Kanagawa, Japan; d Institute for Pathology, University Hospital Cologne, Cologne, Germany; e German Center for Esophageal and Gastric Surgery, Agaplesion Markus Hospital, Frankfurt, Germany; f Department of Visceral Surgery, University Hospital Cologne, Cologne, Germany; g Department of Clinical Genetics, University of Rotterdam, Erasmus University Medical Center, Rotterdam, The Netherlands; h Medical Research Council Clinical Trials Unit at University College London, London, UK; i The Royal Marsden Hospital NHS Foundation Trust, London and Surrey, UK; j Department of Surgery, Royal Marsden National Health Services Foundation Trust, London, UK

**Keywords:** Oesophageal cancer, Gastric cancer, DNA mismatch repair, Microsatellite instability, Epstein–Barr virus

## Abstract

**Background:**

Oesophageal (OeC) and gastric (GC) cancer patients are treated with similar multimodal therapy and have poor survival. There remains an urgent clinical need to identify biomarkers to individualise patient management and improve outcomes. Therapy with immune checkpoint inhibitors has shown promising results in other cancers. Proposed biomarkers to predict potential response to immune checkpoint inhibitors include DNA mismatch repair (MMR) and/or Epstein–Barr virus (EBV) status. The aim of this study was to establish and compare EBV status and MMR status in large multi-centre series of OeC and GC.

**Methods:**

EBV was assessed by EBV-encoded RNA (EBER) *in situ* hybridisation and MMR protein expression by immunohistochemistry (IHC) in 988 OeC and 1213 GC from multiple centres. In a subset of OeC, microsatellite instability (MSI) was tested in parallel with MMR IHC.

**Results:**

Frequency of MMR deficiency (MMRdef) and MSI was low in OeC (0.8% and 0.6%, respectively) compared with GC (10.3%). None of the OeCs were EBER positive in contrast to 4.8% EBER positive GC. EBV positive GC patients were younger (*p* = 0.01), more often male (*p* = 0.001) and had a better overall survival (*p* = 0.012). MMRdef GC patients were older (*p* = 0.001) and showed more often intestinal-type histology (*p* = 0.022).

**Conclusions:**

This is the largest study to date indicating that EBV and MMRdef do not play a role in OeC carcinogenesis in contrast to GC. The potential clinical usefulness of determining MMRdef/EBV status to screen patients for eligibility for immune-targeting therapy differs between OeC and GC patients.

## Highlights

• In oesophageal cancer (OeC) the frequency of mismatch repair deficiency/microsatellite instability (MSI) was extremely low.
• Epstein-Barr Virus infection does not play a role in OeC carcinogenesis.
• Determination of EBV/MSI status for immunotherapy eligibility cannot be recommended for OeC patients.
• Future trials involving immunotherapy require disease specific design and selection criteria.

## 1 Introduction

Oesophageal cancer (OeC) and gastric cancer (GC) are the eighth and fifth most common cancer worldwide, respectively, with an estimated total of 1,407,000 new cases and 1,123,000 deaths in 2012 [1]. The two main histological OeC subtypes are squamous cell carcinoma (SqC) and adenocarcinoma (AdC). The vast majority of GCs are adenocarcinomas.

In Europe, the standard of care for OeC and GC patients with locally advanced resectable disease is chemotherapy or chemoradiotherapy, followed by surgery [2], [3]. GC patients receive perioperative platinum/fluorouracil based chemotherapy. For OeC, patients with SqC are treated with preoperative chemoradiotherapy with carboplatin/paclitaxel. Patients with AdC receive perioperative platinum/fluorouracil or preoperative chemoradiotherapy. Nevertheless, survival remains poor, with 5-year overall survival between 36 and 47% [4], [5].

To date, few targeted therapy options are available to OeC/GC patients with metastatic disease: trastuzumab for HER2 positive disease [6] and ramucirumab, a VEGFR-2 antagonist without biomarker based patient selection [7], [8]. All other trials evaluating receptor tyrosine kinase or downstream signalling inhibitors in OeC/GC were unable to show a survival benefit [9]. There remains an urgent clinical need to identify biomarkers to individualise and improve OeC/GC patient management.

DNA mismatch repair (MMR) has been used as a predictive biomarker for PD1 inhibitor therapy response in multiple different cancer types, including colorectal cancer [10]. Evidence of Epstein-Barr virus (EBV) infection has been proposed as a potential marker for response to PD1/PDL1 inhibitors in GC [11]. Pembrolizumab, an antibody against PD1, was approved by the FDA for the treatment of unresectable or metastatic solid tumours, including OeC and GC, with mismatch repair deficiency (MMRdef) or microsatellite instability (MSI)-High [12].

The potential of immunotherapy in OeC was shown recently in phase 2 trials in non-selected oesophageal SqC and GC patients treated with nivolumab, a monocolonal antibody inhibiting PD1, in second line treatment [13], [14] and in a phase 3 trial in heavily pretreated non-selected Asian GC patients [15]. Furthermore, recent results from the phase 1b trials in patients with PD-L1 expressing OeC (KEYNOTE-028) and GC (KEYNOTE-012), showed promising activity of pembrolizumab in the metastatic setting [16], [17]. In metastatic colorectal cancer, a phase 2 study demonstrated the clinical benefit of pembrolizumab in patients with MMRdef [18].

In addition to the potential role of MMR proteins in selecting patients for immunotherapy, MMRdef has shown prognostic value [19] and seems to predict a poor response to fluorouracil based chemotherapy in colorectal cancer [20], [21]. It has been shown recently in MAGIC trial patients, that gastro-oesophageal cancer patients with MMRdef/MSI tumours treated with surgery alone survived longer compared with those treated with perioperative cytotoxic chemotherapy [22]. In OeC, MLH1 and MSH2 deficiency has been shown to be associated with poor prognosis in small series of SqC [23].

To date, the frequency of MMRdef/MSI in OeC cancer remains unclear because of the small sample size of studies. The reported frequency of MSI-High (MSI-H) ranges from 0 to 27%, but a number of previous studies did not distinguish between MSI-H and MSI-Low (MSI-L) (for an overview of all published studies on MMR and MSI in OeC, see Table 1). The recent study by The Cancer Genome Atlas (TCGA) did not find MSI in any of the 162 OeC [24]. With respect to the frequency of EBV infection in OeC, the majority of previous studies investigated SqC using different methodology, included relatively small number of patients and reported a frequency of EBV positivity from 0 to 36% (for an overview of all published studies on EBV in OeC, see Table 2). Thus, neither MSI/MMRdef nor EBV status has been investigated in large series of OeC using the same methodology and relating results to clinicopathological variables and patient survival.

**Table 1:** Summary of published literature relating to the frequency of mismatch repair deficiency and microsatellite instability in oesophageal cancer.

Authors	Year	Oesophageal cancer type	Total n	MMRdef n (%)	MSI-High n (%)	Method
TCGA [24]	2017	SqC	90	NI	0	PCR
		AdC	70		0	
		undiff	2		0	
Pandilla *et al.*[57]	2013	SqC	60	NI	6 (10)	PCR
		AdC	30		2 (7)	
Farris *et al.*[38]	2011	SqC	76	5 (7)	5 (7)	IHC, PCR
Vasavi *et al.*[44]	2010	SqC	45	NI	12 (27)	PCR
		AdC	5		1 (20)	
Matsumoto *et al.*[58]	2007	SqC	62	NI	5 (8)	PCR
Falkenback *et al.*[48]	2005	AdC	59	2 (3)	2 (3)	IHC, PCR
Naidoo *et al.*[59]	2005	SqC	100	NI	5 (5)^a^	PCR
Uehara *et al.*[23]	2005	SqC	122	49 (40)	6 (5)^a^	IHC
Evans *et al.*[47]	2004	AdC	27	6 (22)	0	IHC, PCR
Araki *et al.*[45]	2004	SqC	100	NI	0	PCR
Hayashi *et al.*[60]	2003	SqC	30	NI	1 (3)	PCR
Ikeguchi *et al.*[46]	1999	SqC	20	NI	1 (5)^a^	PCR
Wu *et al.*[61]	1998	SqC	92	NI	5 (5)^a^	PCR
Muzeau *et al.*[43]	1997	SqC	20	NI	0	PCR
		AdC	26		0	
Gleeson *et al.*[62]	1996	AdC	17	NI	1 (17)	PCR
Keller *et al.*[63]	1995	AdC	15	NI	2 (13)^a^	PCR
Ogasawara *et al.*[64]	1995	SqC	35	NI	21 (60)^a^	PCR
Meltzer *et al.*[65]	1994	SqC	42	NI	1 (2)^a^	PCR
		AdC	36		2 (6)^a^	

Abbreviations: AdC, adenocarcinoma; SqC, squamous cell carcinoma; MMRdef, mismatch repair deficiency; MSI, microsatellite instability; PCR, polymerase chain reaction; IHC, immunohistochemistry; NI, not investigated; undiff, undifferentiated.

a no distinction made between MSI-High and MSI-Low.

**Table 2:** Summary of published literature relating to the frequency of Epstein-Barr virus in oesophageal cancer.

Reference	Year	Oesophageal cancer type	Total n	EBV positive n (%)	Method
TCGA [24]	2017	SqC	90	0	Whole-exome sequencing
		AdC	70	0	
		undiff	2	0	
Genitsch *et al.*[34]	2015	AdC	118	0	EBER ISH
Farris *et al.*[38]	2011	AdC	76	1 (1)	EBER ISH
Sunpaweravong *et al.*[36]	2005	SqC	104	0	EBER ISH
Wu *et al.*[39]	2005	SqC	151	6 (4)	EBER ISH
		undiff	13	4 (31)	
Awerkiew *et al.*[40]	2003	SqC	23	8 (35)	PCR
		AdC	14	5 (36)	
Yanai *et al.*[33]	2003	SqC	34	0	EBER ISH, PCR
Mizobuchi *et al.*[37]	1997	SqC	41	0	PCR
Wang *et al.*[35]	1999	SqC	51	0	EBER ISH, PCR
Wang *et al.*[41]	1999	SqC	31	11 (36)	EBER ISH, PCR

Abbreviations: AdC, adenocarcinoma; SqC, squamous cell carcinoma; EBER ISH, EBV-encoded RNA *in situ* hybridisation; PCR, polymerase chain reaction; undiff, undifferentiated.

The aim of this multi-centre study was to establish the EBV and MMR/MSI status in 988 OeC, including patients from the Medical Research Council (MRC) Oe02 trial [25], from Leeds (UK) and from Cologne (Germany) and relate the results to clinicopathological variables, survival and treatment interaction (preoperative chemo(radio)therapy). As patients with resectable OeC and GC are often treated using similar neoadjuvant therapy regimens and recruited into the same clinical trials across different countries or continents, we compared the frequency of EBV positivity and MMRdef in OeC with that of 1213 GC from Leeds (UK) and Yokohama (Japan).

## 2 Material and methods

### 2.1 General remarks

The definition whether a tumour is a gastric or oesophageal cancer is dependent on the macroscopic location of the bulk/epicentre of the tumour with respect to the gastro-oesophageal junction. Macroscopic images were not available to us for review as part of this study with the exception of the Japanese gastric cancer cases. In contrast to our Japanese colleagues who classify tumours as oesophageal, junctional or gastric, all other pathologists using the TNM classification categorise tumours as being either oesophageal or gastric. We therefore reviewed the macroscopic images from the Japanese junctional cancers to classify them as either oesophageal or gastric according to TNM rules. For all other cases, we have used the classification of the originally reporting pathologist.

### 2.2 Oesophageal cancer cohorts

#### 2.2.1 UK MRC Oe02 trial

The Oe02 trial was a multi-centre phase 3 trial comparing preoperative chemotherapy (cisplatin + 5-fluorouracil) followed by surgery (CS group) to surgery alone (S group) in 802 OeC patients with locally advanced resectable disease, recruited from March 1992 to June 1998. Paraffin blocks of the resected primary tumour were collected retrospectively, and material from 443 patients was available for the present study (CS n = 212, S n = 231). Clinicopathological data which could not be established during the central pathology review were retrieved from pathology reports and the clinical trial database. The study was approved by the South East Research Ethics committee, London, UK, REC reference: 07/H1102/111.

#### 2.2.2 Leeds Teaching Hospitals NHS Trust (LTHT), UK

The LTHT cohort included 223 OeC patients who underwent potentially curative surgery at the Department of Surgery, Leeds General Infirmary (Leeds, UK), between 1986 and 2006. A total of 83 patients had preoperative chemotherapy. Clinical and pathological data were retrieved from pathology reports, electronic patient hospital records and the Northern and Yorkshire Cancer Registry. The study was approved by the Leeds Research Ethics Committee (LREC No. CA01/122).

#### 2.2.3 University Hospital Cologne (UHC), Germany

The UHC cohort included 322 OeC patients who underwent potentially curative surgery at the Department of Visceral Surgery, University of Cologne (Cologne, Germany), between 1999 and 2013. A total of 197 patients had preoperative chemotherapy. Clinical and pathological data were retrieved from pathology reports and electronic patient hospital records. The study was approved by the Ethics Committee at the University Hospital, Cologne (reference number: 09-232).

### 2.3 Gastric cancer cohorts

#### 2.3.1 Leeds Teaching Hospitals NHS Trust, UK

The GC LTHT cohort included 799 patients who underwent potentially curative surgery at the Department of Surgery, Leeds General Infirmary (Leeds, UK) between 1970 and 2004. Eleven patients had preoperative chemotherapy. Demographical, clinical and pathological data were retrieved from pathological reports, electronic patient hospital records and the Northern and Yorkshire Cancer Registry. The study was approved by the Leeds Research Ethics Committee (LREC No. CA01/122).

#### 2.3.2 Kanagawa Cancer Center Hospital (KCCH), Yokohama, Japan

The KCCH cohort included 414 patients with stage II-IV GC who underwent potentially curative surgery at the Kanagawa Cancer Center Hospital (Yokohama, Japan) between 2001 and 2010. None of the patients had preoperative chemotherapy, 202 patients were treated with chemotherapy after surgery. Demographical, clinical and pathological data were retrieved from pathological reports and patient hospital records. The study was approved by the Local Research Ethics Committee.

### 2.4 Methods

#### 2.4.1 Cancer staging and histological subtyping

pT and pN stage was reported according to the Union for International Cancer control 6th and 7th edition of the TNM classification for OeC and GC, respectively.

The histological subtype of adenocarcinomas was established based on Lauren's classification [26]. According to Lauren's classification, signet-ring cell GCs were classified as diffuse-type cancer. As there is no category for mucinous cancers in the Lauren classification, such cancers were classified together with the mixed-type cancers which we used as a category for truly mixed-type cancers and cancers with indeterminate phenotype like the mucinous cancers. The histology type of the case, as stated in the pathology report, was used for statistical analyses.

#### 2.4.2 Tissue microarray construction

Slides from all resection specimens were reviewed and a block with the highest tumour cell density was selected for tissue microarray (TMA) construction and/or marked for microdissection for DNA extraction (see below). The areas selected were representative of the overall histology of the case. The LTHT, KCCH, and Oe02 trial cases were reviewed by HG, LH and GH, together with local pathologists. The UHC cases were reviewed by AQ. A total of 962 OeCs (417, 223 and 322 patients from the Oe02, LTHT and UHC cohorts, respectively) and 1213 GCs (799 and 414 patients from LTHT and KCCH cohorts, respectively) were included in TMAs. TMA construction from the LTHT (OeC and GC) and Oe02 patient cohorts was performed using 0.6 mm tissue cores; 1.2 mm and 1 mm tissue cores were used for the UHC and KCCH cohorts, respectively.

#### 2.4.3 Immunohistochemistry for mismatch repair proteins

MMR immunohistochemistry (IHC) data from previous studies were available for 230 KCCH [27] and 175 LTHT [28] GCs. Additional 184 KCCH and 624 LTHT GCs were stained as part of the present study.

TMA sections from the Oe02 trial cohort were stained for MLH1, MSH2, MSH6, PMS2, from the UHC cohort for MLH1, MSH2 and MSH6 and from the KCCH and LTHT cohort (OeC and GC) for MLH1 and MSH2. For details on antigen retrieval, primary antibodies, detection system, staining protocols see Table 1 in the supplementary material. For all cohorts, 3,3′-Diaminobenzidine (DAB) was used as a chromogen and haematoxylin as a counterstain.

A case was classified as MMR deficient (MMRdef) if tumour cell nuclei were negative for one or more MMR proteins in the presence of positively stained lymphocytes or fibroblasts as internal control. In the Oe02 trial cohort, 12 cases were negative for at least one MMR protein without positive internal controls on the TMA. For these cases, IHC was repeated on full sections. A case was classified as MMR proficient (MMRprof) if tumour cell nuclei, irrespective of the number or intensity, were positive for all MMR proteins tested.

#### 2.4.4 EBV RNA *in situ* hybridisation

EBV data from a previous study were available for 437 LTHT and 216 KCCH GC [28]. Additional 362 LTHT and 198 KCCH GCs were stained as part of the present study. EBV status was determined on TMAs in the LTHT (OeC and GC), Oe02 and KCCH cohorts by EBV-encoded RNA (EBER) *in situ* hybridisation as previously described [29]. In the UHC cohort, a fluorescein-conjugated oligonucleotide probe in conjunction with a monoclonal anti-fluorescein antibody and DAB as chromogen (Leica Biosystems, Wetzlar, Germany) was used according to the instructions of the manufacturer. EBV positivity was defined as presence of staining in tumour cell nuclei, irrespective of the number of nuclei or intensity.

#### 2.4.5 DNA extraction

DNA was extracted using a protocol based on the QIAmp DNA Micro Kit (Qiagen, Hilden, Germany) as previously described [30]. DNA concentration was measured by ND-100 Spectrophotometer (Labtech International) and adjusted to a final concentration of 1 ng/μl.

#### 2.4.6 Assessment of microsatellite instability

The MSI Analysis System, version 1.2 (Promega, Southampton, UK), was used for the detection of MSI in 419 Oe02 patients. This kit allows the simultaneous evaluation of 5 fluorescently labelled MSI markers: BAT-25, BAT-26, NR-21, NR-24 and MONO-27. PCR products were analysed using a 3100-Avant genetic analyser (Applied Biosystems, California, USA) as previously described [27]. Instability in two or more microsatellite loci was categorised as MSI-high (MSI-H) and in a single loci as MSI-low (MSI-L). Absence of MSI in all 5 markers and MSI-L were grouped as microsatellite stable (MSS) for further analyses following current guidelines [31].

#### 2.4.7 Statistical analyses

All statistical analyses were performed using SPSS version 23 software (SPSS Inc., Chicago, III). The relationship between EBV or MMR status and clinicopathological variables (age, gender, depth of invasion (pT), lymph node status (pN), Lauren classification and neoadjuvant treatment) were assessed using chi-squared for categorical variables and Mann-Whitney U for continuous variables. LTHT and KCCH GC data were combined for the analysis of the relationship between EBV or MMR status and overall 5-year survival and differences were assessed using the log rank test. P values less than 0.05 were considered significant.

## 3 Results

### 3.1 EBV status

EBV data were available from 928 OeC patients (LTHT n = 223; Oe02 n = 383; UHC n = 322) and 1178 GC patients (LTHT n = 768; KCCH n = 410). All OeC were EBV negative. A total of 56 (4.8%) GC were EBV positive (LTHT: n = 30 [3.9%], KCCH: n = 26 [6.3%]). Supplementary figure 1 illustrates EBV staining in GC.

### 3.2 Microsatellite status and mismatch repair protein expression

MSI data were available from 362 OeC from the Oe02 cohort. A total of 57 (13.6%) cases had to be excluded due to repeated technical failures. A total of 356 (98.3%) OeC patients were classified as MSS, 4 (1.1%) OeC as MSI-L (3 AdC and 1 SqC) and 2 (0.6%) OeC as MSI-H (both AdC). Supplementary figure 2 shows a typical capillary electrophoresis output for a MSI-H OeC and a MSS OeC. For 306 patients, MMR IHC (MLH1, MSH2, MSH6 and PMS2) data and MSI testing results were available and showed 99.0% concordant results. We therefore decided to only use IHC for the remaining cohorts.

MMR expression data were available from a total of 916 OeC (LTHT n = 220; Oe02 n = 374; UHC n = 322). A sum of 43 (10.3%) and 3 (1.3%) OeC from the Oe02 and LTHT cohorts, respectively, were excluded due to technical failures. Seven (0.8%) OeC (5 AdC and 2 SqC) were classified as MMRdef (LTHT: 3 (1.4%) MLH1 deficient, Oe02: 1 (0.3%) MSH2 deficient, UHC: 3 (0.9%) MLH1 deficient). Patient clinicopathological variables and MMR status for OeC are summarised in Table 3. Owing to the very small number of MMRdef in OeC, it was not feasible to perform any statistical analysis with clinicopathological data or survival.

**Table 3:** Mismatch repair status and clinicopathological variables in patients with oesophageal cancer.

Clinicopathological variables		Mismatch repair proficient						Mismatch repair deficient					
		LTHT		Oe02		UHC		LTHT		Oe02		UHC	
		*n*	%	*n*	%	*n*	%	*n*	%	*n*	%	*n*	%
Sex	Male	137	63.1	294	78.8	287	89.9	2	66.7			3	100
	Female	80	36.9	79	21.2	32	10.1	1	33.3	1	100		
(y)pT(6)	T0	2	0.9			3	0.9						
	T1	32	14.7	27	7.2	63	19.7					1	33.3
	T2	38	17.5	36	9.7	63	19.7	1	33.3				
	T3	136	62.7	301	80.7	185	58	2	66.7	1	100	2	66.7
	T4	9	4.1	9	2.4	5	1.6						
(y)pN(6)	N0	83	38.2	123	33	122	38.2			1	100	3	100
	N1	133	61.3	250	67	197	61.8	3	100				
	unknown	1	0.5										
Histological type	Adenocarcinoma	165	76	275	73.7	319	100	2	66.7			3	100
	Squamous cell carcinoma	49	22.6	87	23.3			1	33.3	1	100		
	Other	3	1.4	11	2.9								
Neoadjuvant treatment	Yes	80	36.9	177	47.5	194	60.8	2	66.7	1	100	2	66.7
	No	133	61.3	196	52.5	125	39.2	1	33.3			1	33.3
	unknown	4	1.8										

Abbreviations: LTHT, Leeds Teaching Hospital Trust; Oe02, oesophageal cancer trial 02 [25]; UHC, University Hospital Cologne.

MMR protein expression data were available from 1098 GC (LTHT n = 702; KCCH n = 396). A total of 113 (10.3%) cases were classified as MMRdef (LTHT: 70 (10.0%), KCCH: 43 (10.9%)). Supplementary figure 3 illustrates MMR protein expression in a MMRdef GC.

For 1063 GCs, both EBV and MMR data were available. A single GC from the LTHT cohort was MMRdef and EBV positive. This patient was male, 67 years old at the time of diagnosis and survived 17 years despite having an advanced intestinal-type GC (pT4, pN3) in the resected specimen.

### 3.3 Relationship of EBV status and MMR status with clinicopathological variables in patients with gastric cancer

Patients with EBV positive GC were younger (median [range] age EBV positive GC: 63 years (32–89 years) versus 68 years (14–96 years) in EBV negative GC, *p* = 0.01). A total of 48 (85.7%) patients with EBV positive GC were male compared with 8 (14.3%) of female patients (*p* = 0.001). EBV positive GC patients had a better overall 5-year survival compared with EBV negative GC patients (60.7% versus 41.7%; hazard ratio 1.72, 95% confidence interval 1.12–2.63 [*p* = 0.012]).

Patients with MMRdef GC were older (median [range] age MMRdef GC: 71 years [51–90 years] versus 68 years [24–96 years] in MMRprof GC, *p* = 0.001). A total of 77 (69.4%) MMRdef GC had intestinal-type histology compared with 20 (18.0%) with diffuse-type histology (*p* = 0.022). There was no difference in overall survival between MMRdef and MMRprof GCs (*p* = 0.383). There was no relationship with any other clinicopathological variables (Table 4).

**Table 4:** Comparison of mismatch repair and EBV status with clinicopathological variables in patients with gastric cancer.

Clinicopathological variables		Mismatch repair proficient						Mismatch repair deficient						p value	EBV negative						EBV positive						p value
		LTHT		KCCH		Total		LTHT		KCCH		Total			LTHT		KCCH		Total		LTHT		KCCH		Total		
		n	%	n	%	n	%	n	%	n	%	n	%		n	%	n	%	n	%	n	%	n	%	n	%	
Gender	Male	415	59	250	63	665	61	42	6	33	8	75	7	0.761	456	59	273	67	729	62	26	3	22	5	48	4	**0.001**
	Female	214	30	102	26	316	29	28	4	10	3	38	3		281	37	110	27	391	33	4	1	4	1	8	1	
	Unknown	3	0	1	0	4	0								1	0	1	0	2	0							
(y)pT(7)	T1	83	12	34	9	117	11	5	1	3	1	8	1	0.074	105	14	37	9	142	12	4	1	2	0	6	1	0.794
	T2	69	10	52	13	121	11	2	0	5	1	7	1		75	10	58	14	133	11	5	1	4	1	9	1	
	T3	179	25	52	13	231	21	26	4	3	1	29	3		210	27	52	13	262	22	9	1	3	1	12	1	
	T4	301	43	214	54	515	47	37	5	32	8	69	6		348	45	236	58	584	50	12	2	17	4	29	2	
	Unknown			1	0	1	0										1	0	1	0							
(y)pN(7)	N0	206	29	70	18	276	25	22	3	13	3	35	3	0.722	242	32	82	20	324	28	13	2	4	1	17	1	0.931
	N1	123	18	80	20	203	18	19	3	6	2	25	2		155	20	83	20	238	20	6	1	5	1	11	1	
	N2	146	21	91	23	237	22	14	2	8	2	22	2		152	20	96	23	248	21	7	1	7	2	14	1	
	N3	156	22	111	28	267	24	15	2	16	4	31	3		189	25	122	30	311	26	4	1	10	2	14	1	
	Unknown	1	0	1	0	2	0										1	0	1	0							
Lauren classification	Intestinal	403	57	181	46	584	53	49	7	28	7	77	7	**0.022**	461	60	204	50	665	56	20	3	15	4	35	3	0.919
	Diffuse	145	21	154	39	299	27	10	1	10	3	20	2		185	24	156	38	341	29	6	1	10	2	16	1	
	Mucinous/mixed	82	12	15	4	97	9	11	2	3	1	14	1		90	12	17	4	107	9	4	1	1	0	5	0	
	Unknown	2	0	3	1	5	0			2	1				2	0	7	2	9	1							
Neoadjuvant treatment	Yes	8	1	177	45	185	17	1	0	16	4	17	2	0.305	11	1	185	45	196	17			13	3	13	1	0.293
	No	624	89	164	41	788	72	69	10	27	7	96	9		727	95	185	45	912	77	30	4	13	3	43	4	
	Unknown			12	3	12	1										14	3	14	1							

Abbreviations: KCCH, Kanagawa Cancer Center Hospital; LTHT, Leeds Teaching Hospital Trust.

p-Values in bold are considered statistically significant.

A summary of the EBV, MMR and MSI status in each cohort is provided in Table 5.

**Table 5:** Summary of EBV, mismatch repair and microsatellite instability status in oesophageal and gastric cancer.

Molecular characteristics		OeC						GC			
		Oe02		LTHT		UHC		LTHT		KCCH	
		n = 443	%	n = 223	%	n = 322	%	n = 768	%	n = 410	%
EBV	Negative	383	100	223	100	322	100	738	96	384	94
	Positive	0	0	0	0	0	0	30	4	26	6
MMR	Proficient	373	100	217	99	319	99	632	90	353	89
	Deficient	1	0	3	1	3	1	70	10	43	11
Microsatellite	Stable	356	98	NI		NI		NI		NI	
	Instable-Low	4	1	NI		NI		NI		NI	
	Instable-High	2	1	NI		NI		NI		NI	

Abbreviations: EBV, Epstein-Barr Virus; GC, gastric cancer; KCCH, Kanagawa Cancer Center Hospital; LTHT, Leeds Teaching Hospitals NHS Trust; MMR, mismatch repair; MSI, microsatellite instability; MSS, microsatellite stable; OeC, oesophageal cancer; UHC, University Hospital Cologne; NI, not investigated.

## 4 Discussion

This is the largest gastro-oesophageal cancer study to date investigating MMR and EBV status in 988 OeC and 1213 GC. The extremely low frequency of MMR/MSI and lack of EBV infection in OeC relative to GC in our study confirms the recent TCGA results which investigated MSI and EBV in smaller series of 164 OeC [24] and 295 GC [11] using different methodologies.

All OeC were EBV negative which is consistent with the majority of previously published studies [32], [33], [34], [35], [36], [37]. Therefore, we can conclude now that EBV does not play a role in OeC carcinogenesis neither in SqC nor in AdC. A small number of previous studies reported an EBV positivity rate between 1 and 36% in OeC [38], [39], [40], [41]. This discrepancy is most likely related to different potentially less reliable methodology, such as PCR, which would also detect EBV in tumour-infiltrating lymphocytes [33] leading to false positive results. The present study used the generally accepted ‘gold standard’ EBER methodology. In our study, EBV positive GC patients had a significantly better overall survival compared with EBV negative patients which is consistent with results from other studies [42].

In the Oe02 cohort, we detected a very low frequency of MSI-H (0.6%) using the Bethesda microsatellite panel [31]. This result is consistent with the recent smaller TCGA study which found no MSI-H cases in 72 oesophageal AdC [24]. However, our result is in contrast to the literature reporting a frequency of MSI-H in OeC between 0 and 27% in SqC [43], [44], [45], [46] and 0–20% in AdC [22], [38], [43], [44], [47], [48]. Discrepancies in the frequency of MSI-H amongst studies could be related to different definitions of MSI-H [47], as well as differences in location [44] and number of microsatellite loci tested [46]. Recent studies in GC suggest that a mononucleotide and dinucleotide markers different to those included in the so-called Bethesda panel might improve accuracy and sensitivity of MSI testing in GC [49], [50].

There are few small studies reporting a MMRdef frequency of 3–40% in OeC mostly based on IHC of MLH1 and MSH2 [23], [38], [47], [48]. Some of the previous studies scores were based on staining intensity and cell proportions and classifying cases with weak staining and/or low percentages of positively stained tumour cells as MMRdef. Thus, when using our MMR scoring system where a case is classified as MMRprof, irrespective of the number of positive nuclei or staining intensity, the frequency of MMRdef in our study is comparable to previously published studies. Another potential reason for discrepant results in the literature could be the misclassification of AdC with a tumour bulk located in the stomach which extends into the GOJ as OeC. In contrast to the results from the MAGIC trial patients [22], there was no overall survival difference between MMRdef GC and MMRprof GC in our study. This is likely due to differences in disease stage, histological subtypes and age of GC patients in our study.

The frequency of MMRdef and EBV positivity in our GC cohort is consistent with the current literature [51], [52], [53]. As the same methodology was used to stain GC and OeC, our GC results also indirectly support the reliability of the low frequency of MMRdef and EBV in OeC in the present study. Furthermore, our results are comparable with results from a smaller study in the MAGIC trial patients comparing the frequency of MSI and MMRdef in GC and OeC [22].

Our study has some limitations. First, this is a retrospective study. Second, due to limited tissue availability, we were unable to perform IHC for all four MMR proteins in all cases, and we did not test all cases for MSI. However, evidence in the literature from GC found MMRdef was due to loss of MLH1 in 95.8% of cases, and deficiency in MSH6 and PMS2 was rare [51]. Similarly, a colorectal cancer study reported a positive predictive value and specificity of IHC for MMR proteins of 99.1% and 99.6%, respectively, compared with MSI [54]. Our own study showed that MSI status is in 99.0% of cases concordant with the MMR IHC status. Another potential limitation is our inability to determine the proportion of junctional (GOJ) AdC versus true oesophageal or true gastric AdC which might potentially be clinically relevant. This is related to the fact that detailed pre-chemotherapy endoscopic information regarding the location was not available for most cases. There are very few studies investigating EBV and MMRdef in GOJ cancer with inconsistent results most likely related to low sample sizes [22], [34], [55] or differences in defining the GOJ [56].

Our OeC findings suggest that OeC carcinogenesis is not associated with EBV infection and MMRdef/MSI does not appear to be an important underlying mechanism in OeC, neither SqC nor AdC. The use of EBV and/or MMR/MSI status to determine OeC patient eligibility for immunotherapy or adjuvant cytotoxic therapy cannot be recommended, and there remains the need to find alternative biomarkers for such therapy approaches in this patient population. The difference in the frequency of MMRdef and EBV infection between OeC and GC indicate not only pathophysiological differences in oesophageal and gastric carcinomas but might also have important implications for patient selection for future treatment and study planning. In contrast to the current practice of recruiting patients with GC or OeC into the same trials, trials involving immunotherapy require most likely disease specific different designs and selection criteria for patients with OeC.

## Conflict of interest statement

DC received financial support from AstraZeneca, Amgen, Bayer, Celgene, Merrimack, Merck Serono, MedImmune and Sanofi. WA received financial support from Eli Lilly and Nestle. RL received financial support from Bayer. The remaining authors have no conflicts of interest to declare.

## Figures and Tables

**figs1:**
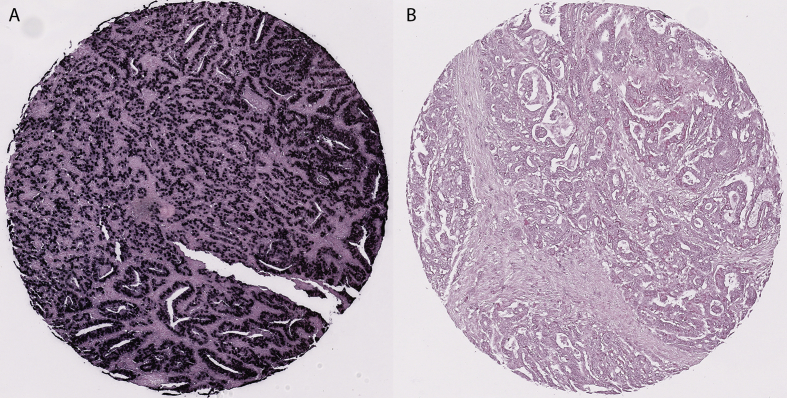


**figs2:**
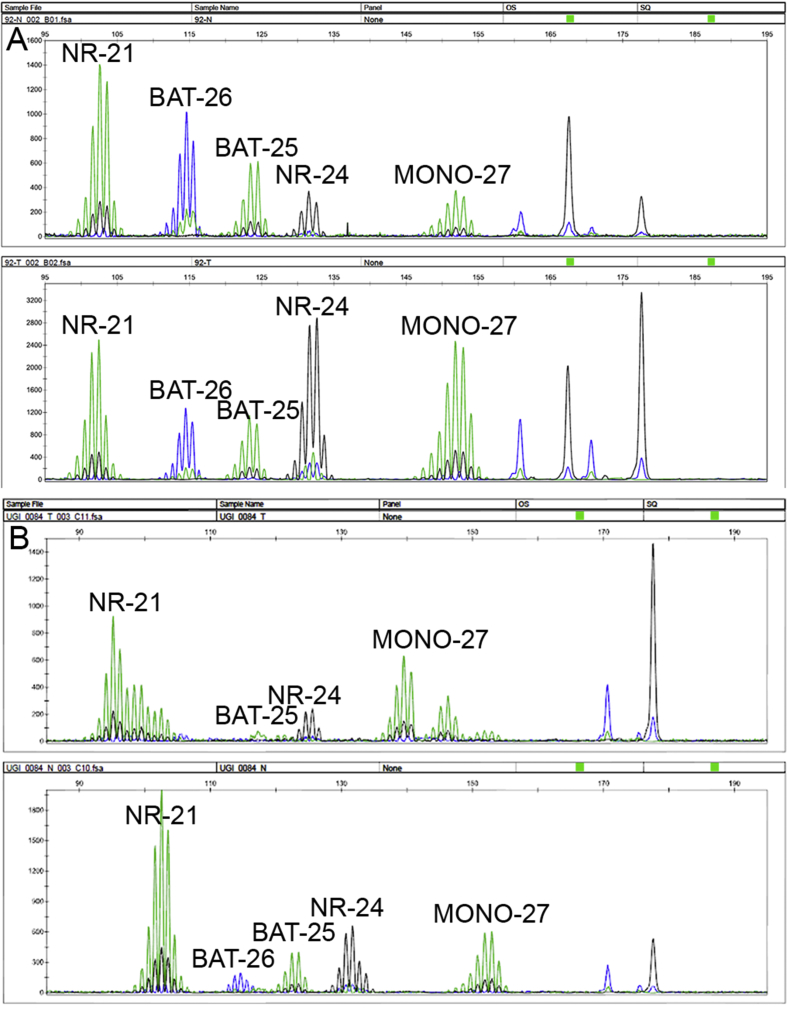


**figs3:**
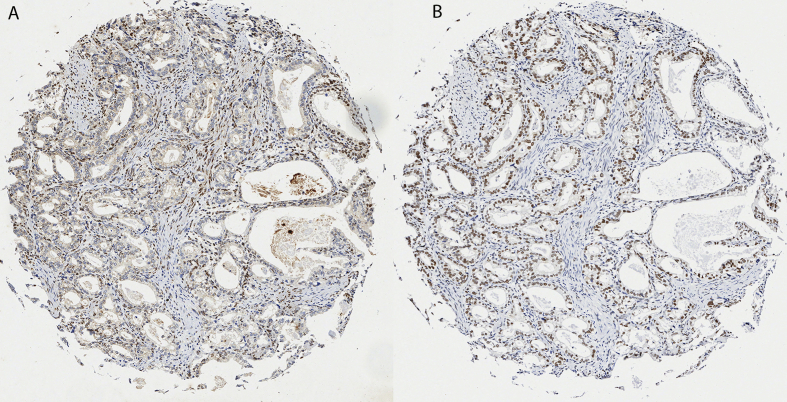


## Appendix A Supplementary data

mmc1

mmc2
