# Supplementary material for: Epstein-Barr virus and mismatch repair deficiency status differ between oesophageal and gastric cancer: A large multi-centre study
Source: Eur J Cancer. 2018 May;94:104–14. doi: 10.1016/j.ejca.2018.02.014 (PMC5914544; doi:10.1016/j.ejca.2018.02.014)
Supplement: mmc1 [file mmc1.docx]

Titles and legends to supplementary figures

Supplementary figure 1:

Epstein Barr Virus-encoded RNA *in situ* hybridization staining. A: EBV positive gastric cancer (black = 5-bromo-4-chloro-3-indolylphosphate and nitroblue tetrazolium, red = counterstain with nuclear fast red). B: EBV negative gastric cancer.

Supplementary figure 2:

MSI analysis output. A: Microsatellite stable oesophageal cancer (upper panel) and matched normal sample (lower panel). B: Microsatellite allele length changes in a microsatellite instable-high oesophageal cancer (upper panel) compared to the matched normal sample (lower panel).

Supplementary figure 3:

Expression of mismatch repair proteins by immunohistochemistry in a mismatch repair deficient gastric cancer. A: Tumour cell nuclei are negative for MLH1, adjacent lymphocytes are positive (brown = DAB, blue = haematoxylin counterstain). B: Tumour cell nuclei are positive staining for MSH2 (brown = DAB, blue = haematoxylin counterstain).
